# A Recurrent Case of Full-Thickness Macular Hole After Successful Closure With Primary Vitrectomy and Epiretinal Proliferation Embedding

**DOI:** 10.7759/cureus.66232

**Published:** 2024-08-05

**Authors:** Shoji Notomi, Yuki Kubo, Keijiro Ishikawa, Satomi Shiose, Sonoda Koh-Hei

**Affiliations:** 1 Ophthalmology, Kyushu University, Fukuoka, JPN

**Keywords:** pars plana vitrectomy, internal limiting membrane peeling, epiretinal proliferation, full-thickness macular hole, surgical retina

## Abstract

Epiretinal proliferation (EP) is thought to be glial cell proliferation arising from the inner retina, seen in cases of lamellar or full-thickness macular holes (FTMH). Embedding EP within the macular hole is considered supportive for FTMH closure and functional recovery. We report a recurrent case of FTMH that was successfully closed after primary vitrectomy with the EP embedding technique. In the primary surgery, internal limiting membrane (ILM) peeling was avoided to reduce the potential risk of retinal nerve fiber layer damage associated with glaucoma. The FTMH was successfully closed, with complete recovery of macular layer structures. However, over one year later, the FTMH reopened, slightly dislocated from the position of the embedded EP scar. The reopened FTMH was closed again after the second surgery using the ILM inverted flap technique. This case indicates that macular hole closure with EP might not sufficiently support the tissue repair of FTMH as a new hole can form if tangential traction of the ILM remains.

## Introduction

A macular hole is a retinal defect located at the center of the fovea and causes significant central vision impairment. It is known to be caused by tangential tractions on the retinal surface, such as vitreoretinal traction of the posterior hyaloid during posterior vitreous detachment (PVD). A full-thickness macular hole (FTMH) involves a defect in all layers of the neurosensory retina, whereas a lamellar macular hole (LMH) presents as a loss of tissue in the inner layers of the retina. 

Epiretinal proliferation (EP) is fibrocellular tissue found on the inner surface of the retina, which can be observed in cases of LMH as well as FTMH. The origin of EP is considered to involve a Müller-cell-driven process that emerges from the inner retinal layers [1]. EP is characterized by optical coherent tomography (OCT) findings such as an isoreflective space-filling material over the retinal surface, a thin highly reflective line, and the absence of apparent tractional properties. Due to its unique morphology that differs from the typical epiretinal membrane (ERM) arising from the posterior hyaloid membrane, several studies investigated its association with surgical outcomes in LMH or FTMH. Notably, previous studies reported that preservation of EP, the so-called EP embedding technique, may be beneficial for the anatomic and functional recovery in macular hole surgery rather than peeling it from the retina [2-4]. This provided a plausible hypothesis that EP originating from Müller glia might support the tissue-repairing process in macular holes.

During surgery for FTMH, peeling of the internal limiting membrane (ILM), the basement membrane of the Müller glia, is often performed as it can provide better anatomical closure of FTMH. However, a number of studies on ILM peeling have cautioned about its potential to damage the inner retina, especially in glaucoma patients as they have more fragile nerve fiber layers than healthy subjects [5]. Hence, it is still debatable how much area of ILM peeling should be performed in FTMH patients complicated with glaucoma. Here, we present a case of FTMH with glaucoma that reopened one year after the primary vitrectomy with EP embedding without ILM peeling.

## Case presentation

**Figure 1:**
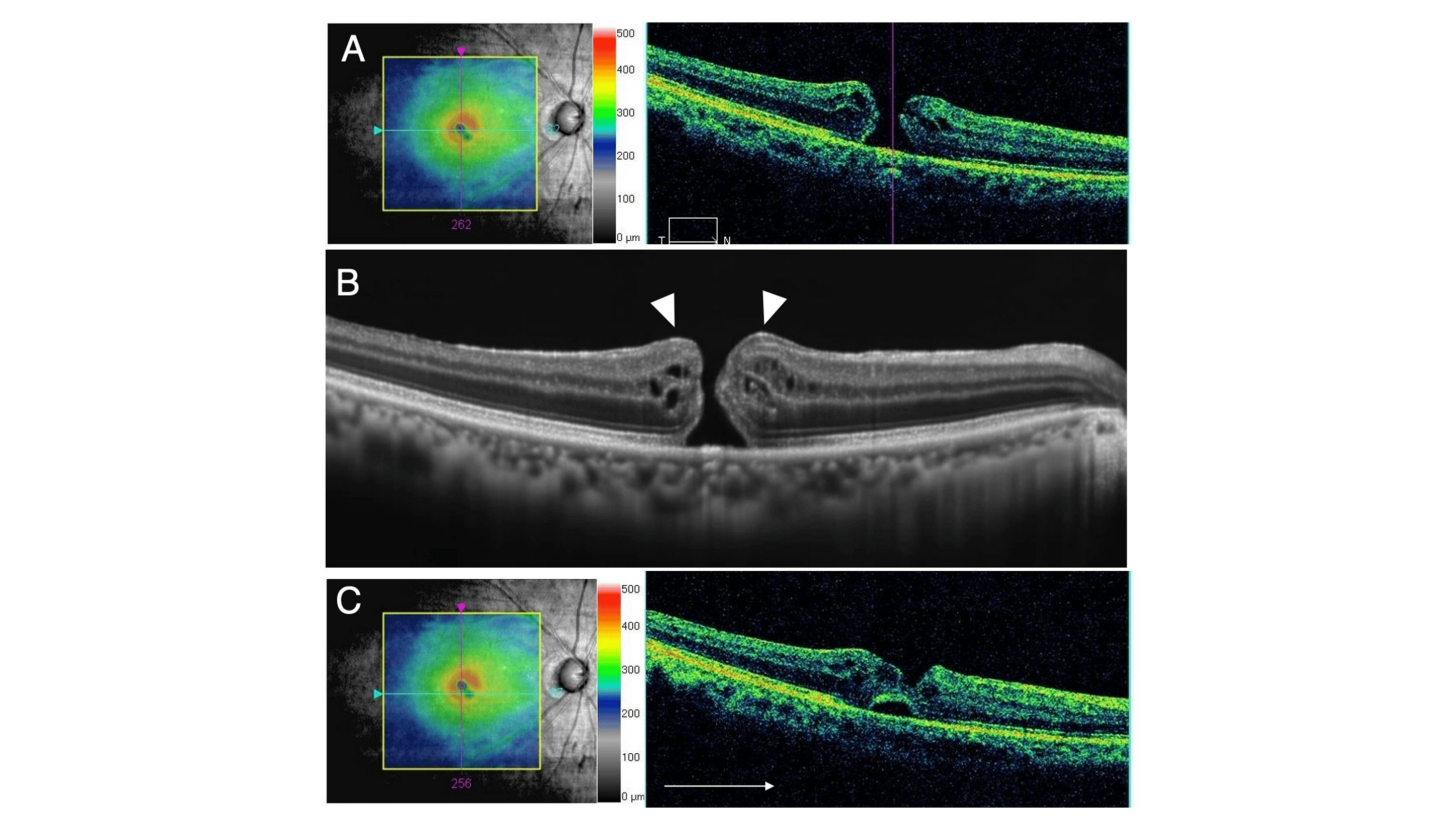
Preoperative OCT images. (A) Preoperative OCT image indicating the location of FTMH at the foveal center. (B) EP is visible on the retinal surface around FTMH (arrowheads). (C) A small dimple of the inner retina was observed on the surface of the inferior nasal of the fovea. OCT, optical coherent tomography; FTMH, full-thickness macular hole; EP, epiretinal proliferation.

A 64-year-old female patient was referred to our hospital for the treatment of FTMH. The duration of symptoms was about four weeks. The best corrected visual acuity (BCVA) was 20/63 and slit-lamp examination revealed moderate cataract in her right eye. The axial length in her right eye was 24.67 mm as measured by a partial coherence interferometer. OCT revealed a stage 4 FTMH with EP and the horizontal minimum linear diameter was 339 microns (Figures 1A, 1B). A small dimple, a defect of the retinal surface, was observed in the inferior nasal of the fovea (Figure 1C).

**Figure 2:**
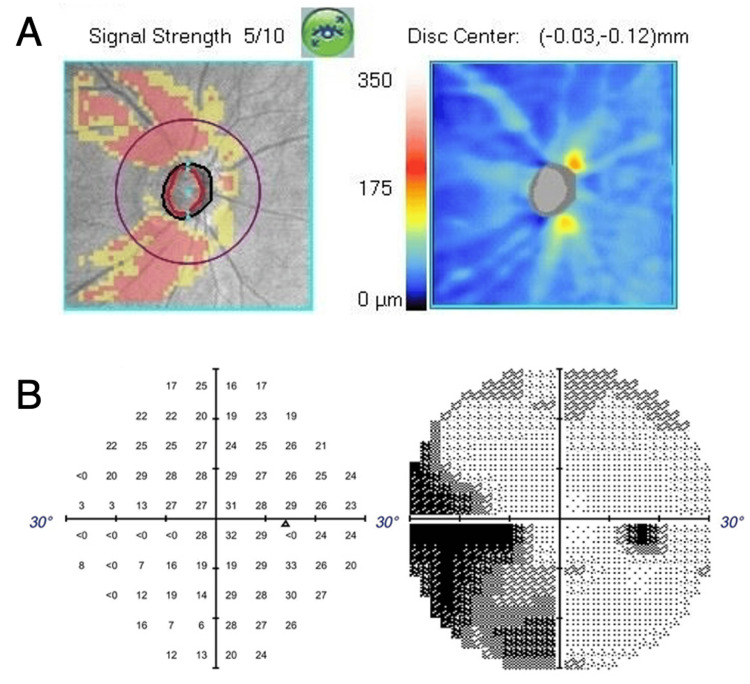
Preoperative OCT of the optic disc and visual field analysis. (A) Preoperative OCT of the optic disc shows a thinning of the temporal rim. (B) The nasal step was observed by a visual field analyzer in the right eye. OCT, optical coherent tomography.

She was receiving topical medication of latanoprost and brimonidine tartrate for open-angle glaucoma. The intraocular pressure in her right eye was 23 mmHg measured by a non-contact tonometer. OCT detected a thinning of the temporal rim (Figure 2A), and the visual field analyzer detected a nasal step in her right eye (Figure 2B).

**Figure 3:**
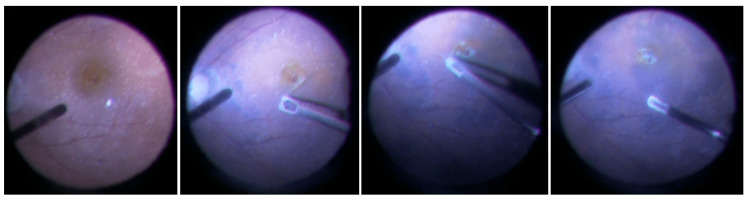
Intraoperative images of EP. Intraoperative images of EP embedding. Perifoveal EP was partially peeled with intraocular forceps and placed on the fovea. EP, epiretinal proliferation.

The primary pars plana vitrectomy (PPV) combined with phacoemulsification and intraocular lens implantation, was performed without ILM peeling to avoid potential damage to the retinal nerve fiber layer in her right eye. During the surgery, a 25-gauge micro-incision PPV was conducted using a Constellation system (Alcon, Vernier, Switzerland). There was a pre-existing PVD. Perimacular EP was peeled off from the retina using intraocular forceps and left attached to the edge of the macular hole (Figure 3). Remaining ILM was confirmed by staining with brilliant blue G. Fluid-air exchange was performed at the end of the surgery, and the vitreous cavity was filled with 20% sulfur hexafluoride (SF6) gas. Topical latanoprost and brimonidine tartrate were continued after the surgery.

**Figure 4:**
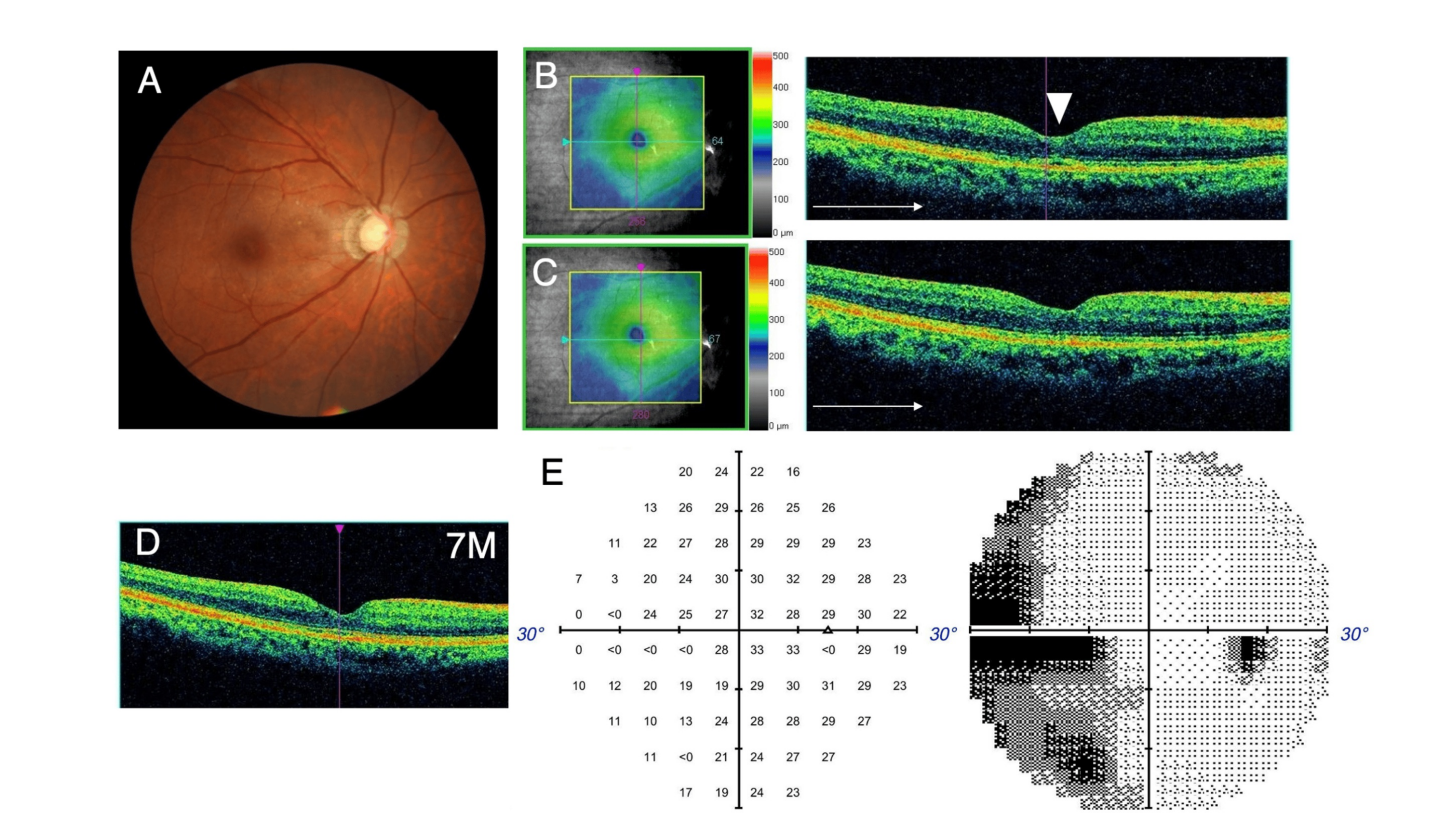
Postoperative fundus photography, OCT images, and visual field analysis. (A, B) Fundus photography and OCT at postoperative three weeks. Embedded EP was visible in the foveal center in OCT at postoperative two months (arrowheads). (C) The inferior-nasal parafoveal small dimple of the retinal surface, which was detected preoperatively, had become smooth after surgery. (D) The macular hole was kept closed at postoperative seven months. The ellipsoid zone line was observed in the fovea. (E) No apparent worsening of visual field was observed at postoperative six months. OCT, optical coherent tomography; EP, epiretinal proliferation.

At postoperative three weeks, the successful closure of FTMH was confirmed by OCT (Figures 4A, 4B). The remaining embedded EP on the foveal center was observed in OCT (Figure 4B). The inferior-nasal parafoveal small defect observed preoperatively had become smooth after surgery (Figure 4C). The BCVA improved up to 20/20. At postoperative seven months, the macular hole was kept closed while retaining a regular ellipsoid zone line (Figure 4D). Visual field analysis at six months postoperatively showed no apparent worsening (Figure 4E).

**Figure 5:**
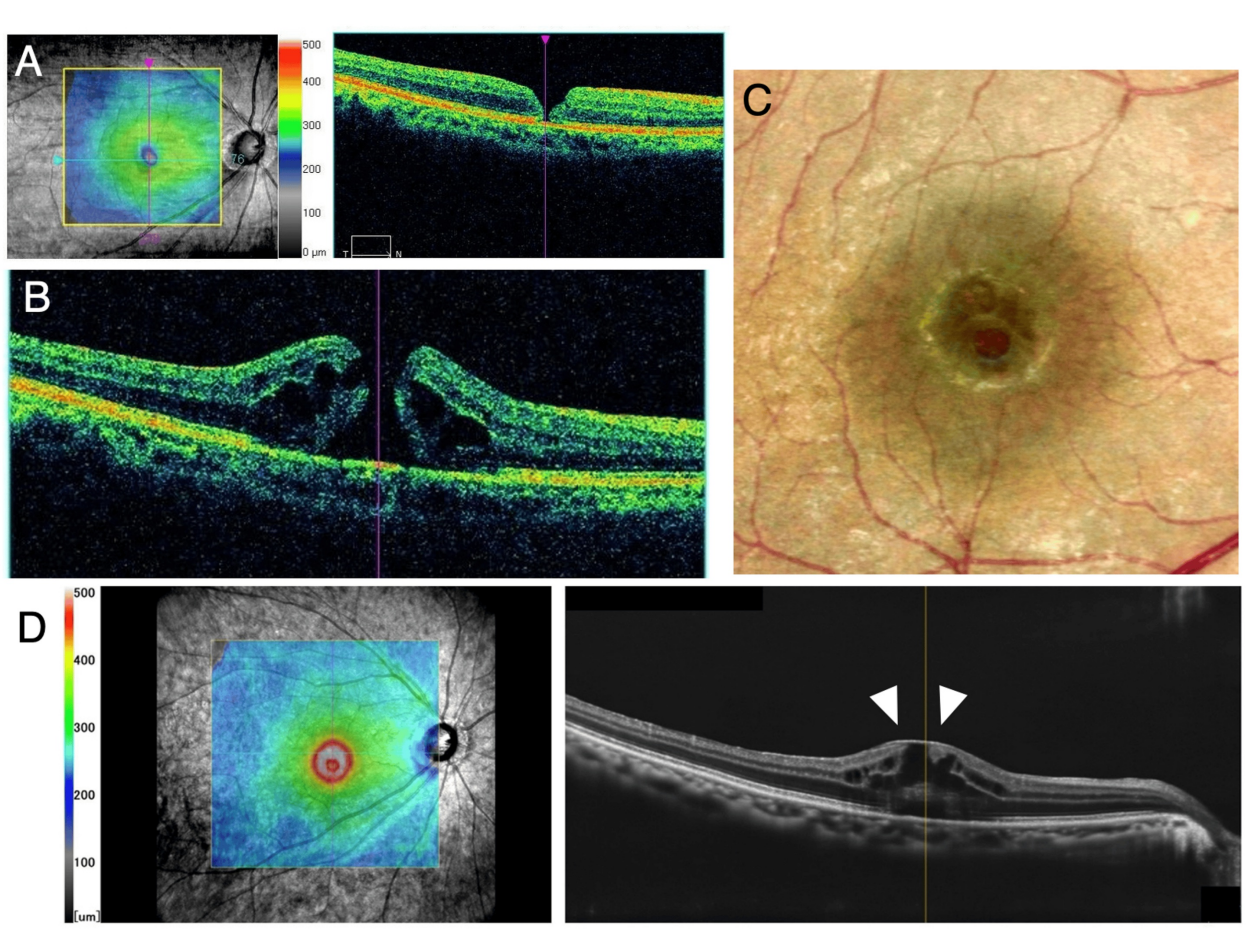
Fundus photography and OCT images of reopened FTMH. (A) Reopening of FTMH was observed in OCT at postoperative 13 months. (B) One month later, fluid cuff and intra-retinal fluid appeared. (C) The reopened FTMH was observed at a slightly inferior position to the scar of EP in fundus photography. (D) The scar of EP was illustrated by OCT as a hyper-reflective line adjacent to the reopened FTMH (arrowheads). OCT, optical coherent tomography; FTMH, full-thickness macular hole; EP, epiretinal proliferation.

However, at postoperative 13 months, a small flat open macular hole was detected by OCT (Figure 5A). The BCVA was 20/30. The location of the re-opened FTMH was observed slightly inferior to the original hole. One month later, fluid cuff and intraretinal edema appeared in OCT (Figure 5B), keeping BCVA 20/30. The recurrent FTMH showed a horizontal minimum linear diameter of 210 microns. The scar of embedded EP was observed at the foveal center, which located superiorly to the reopened FTMH (Figures 5C, 5D).

**Figure 6:**
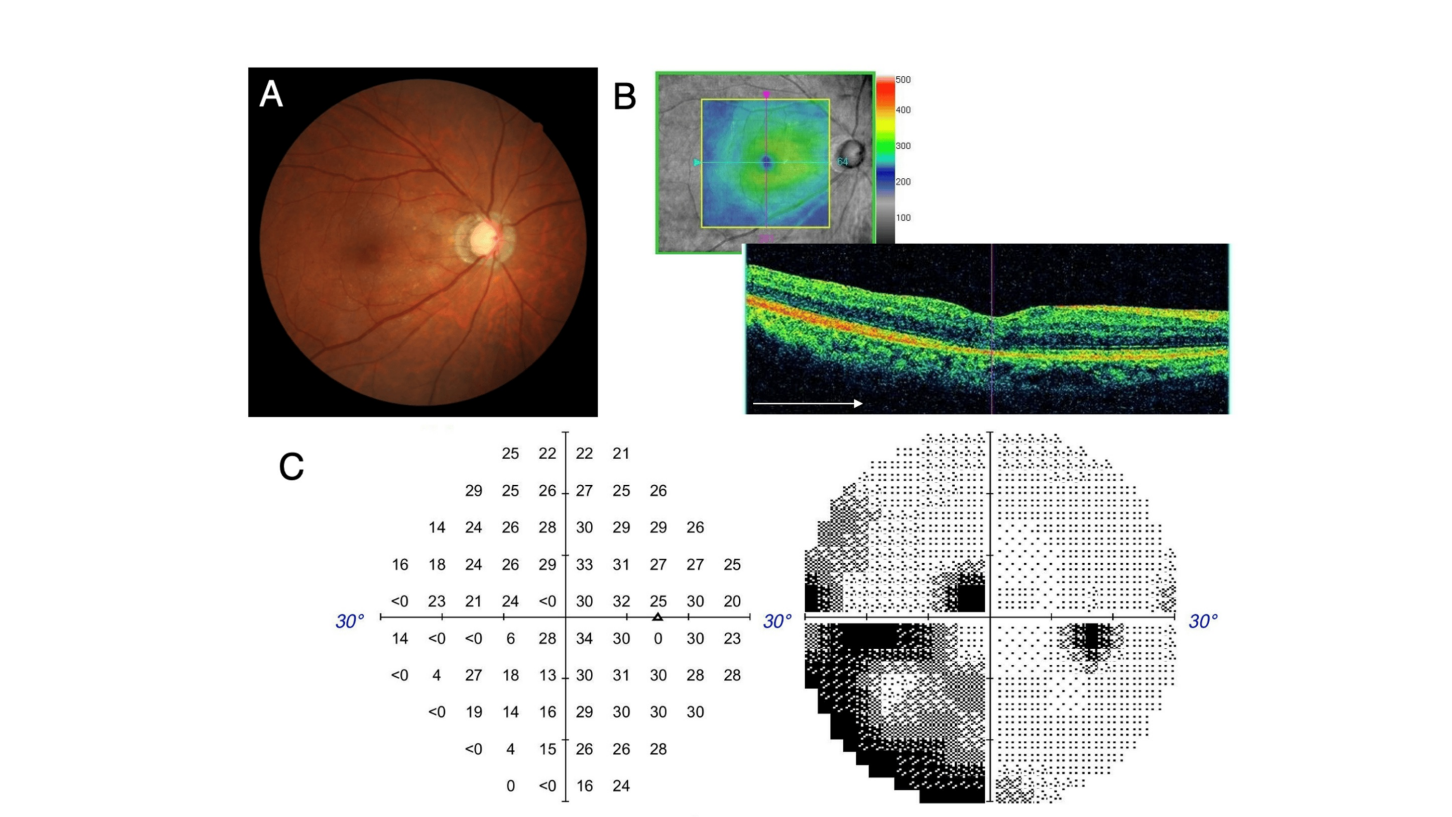
Fundus photography, OCT image, and visual field analysis after the second surgery. (A) Fundus photography. (B) OCT at postoperative two weeks after the second surgery with an inverted ILM flap technique. (C) Visual field analysis at four months after the second surgery detected a paracentral scotoma superior temporarily. The sensitivity of the inferior visual field was not apparently worsened. OCT, optical coherent tomography; ILM, internal limiting membrane.

The second surgery was performed with an ILM inverted flap technique, in which the superior ILM was peeled and inverted to cover FTMH. No apparent residual vitreous cortex was observed on the macular area. The inverted ILM flap was fixed by placing sodium hyaluronate (Shellgan, Santen Pharmaceutical Co., Ltd, Osaka, Japan) on it. Following fluid air exchange, the vitreous cavity was filled with 20% SF6 gas. Topical latanoprost and brimonidine tartrate were continued postoperatively as well. Two weeks after the second surgery, FTMH was successfully closed (Figures 6A, 6B). The BCVA at the final visit, 12 months after the second surgery, was 20/160, and the macular hole was kept closed. Visual field analysis four months after the second surgery detected a paracentral scotoma that may have resulted from the reopening of FTMH. However, the sensitivity of the inferior visual field was not apparently affected by the peeling/inversion of the superior ILM (Figure 6C).

## Discussion

In this case, FTMH was initially closed by primary vitrectomy with EP embedding technique. Postoperative OCT detected EP located in the foveal center, being absorbed gradually over time and leaving fibrous components. Fundus photographs showed an EP-remnant at the site of the closed macular hole. These observations indicated that the migrating Müller cells in EP had become fibrous components over time. Moreover, the reopening of FTMH has appeared slightly dislocated from the scar of EP. This suggests that the tissue repair with EP may not be as robust, compared to the native Müller cell cone [6].

FTMH with EP has been reported to have a higher probability of spontaneous closure than that without EP. Furthermore, the favorable surgical outcomes of FTMH with EP embedding technique suggested a role of EP in promoting tissue repair for hole closure [7,8]. Although ILM peeling is known to be associated with improved rates of hole closure in FTMH with EP [9], there is limited information about the reopening cases of FTMH. Lai et al. reported nine cases with spontaneous hole closure without surgery as well as one case that experienced a reopening after surgery and then spontaneously closed several months later [10]. Hence, we experienced a unique case of FTMH that reopened after surgery and required a second surgery. As our case of reopened FTMH was accompanied by a fluid cuff with smooth edge, we considered that it did not have high expectation of spontaneous closure [11].

The reopening of FTMH occurred over one year later from the first surgery. This could be caused by tangential traction due to the unpeeled ILM [12]. Previous reports have shown that 4.8-9.2% of macular holes reopened after surgery and that ILM peeling can reduce the incidence of macular hole recurrence [13-15]. Also in our case, FTMH reopened at a similar postoperative period. However, it is notable that the position of the reopened FTMH was slightly inferior to the foveal center where the scar of embedded EP was observed. Although tangential traction by the residual ILM might have contributed to the reopening of FTMH, our observations indicated that the reopening of FTMH occurred at a position anatomically different from the original hole closed with embedded EP. Notably, the small inferior-nasal defect of the inner retina was observed preoperatively. Although the defect was anatomically improved after the primary surgery, the preoperative defect might have been associated with the fragility of the fovea, resulting in recurrent FTMH.

For epiretinal membrane surgery, there are ongoing debates on ILM peeling for recurrence prevention because several reports have shown that ILM peeling can lead to anatomical changes such as dissociated optic nerve fiber layer and may even worsen the visual field in glaucoma patients [5]. In our case, there was not an obvious decreased sensitivity in visual field analysis after the primary surgery. Even after the second surgery with ILM peeling of the superior macula, loss of inferior sensitivity was not clearly detected. However, the reopening of FTMH seems to have resulted in the appearance of paracentral scotoma.

## Conclusions

We experienced a recurrent case of full-thickness macular hole (FTMH) that was successfully closed after primary vitrectomy with epiretinal proliferation (EP) embedding technique, but without internal limiting membrane (ILM) peeling to reduce the potential risk of retinal nerve fiber layer damage associated with glaucoma. Even after the successful closure of FTMH with EP embedding, the reopening of FTMH did occur at the adjacent location. Our case suggested that the EP embedding technique may not allow for avoiding ILM peeling in FTMH surgery even if EP embedding might be supportive for tissue repair for closing FTMH. A surgical treatment of EP embedding with ILM peeling or an inverted ILM flap technique may be recommended to reduce the risk of macular hole recurrence in FTMH patients with glaucoma.
